# Prevalence of Iron deficiency anemia in children with liver cirrhosis: A cross-sectional study

**Published:** 2015-07-01

**Authors:** Soheila Zareifar, Seyed Mohsen Dehghani, Najmeh Rahanjam, Mohammad Reza Farahmand Far

**Affiliations:** 1 Pediatric Hematology and Oncology Department, Shiraz University of Medical Sciences, Shiraz, Iran; 2 Gasteroenterology Department, Shiraz University of Medical Sciences, Shiraz, Iran

**Keywords:** Iron deficiency anemia, Cirrhosis, Children

## Abstract

**Background:** Among the many complications reported for cirrhosis, iron deficiency anemia (IDA) has attracted much attention. This type of anemia, in contrast to other types of anemia, is easy to treat prophylactically, but if left untreated can lead to a poor quality of life. The aim of this study was to estimate the hemoglobin and serum iron levels among patients with liver cirrhosis for the early diagnosis of IDA and to avoid unnecessary testing and iron supplementation.

**Subjects and Methods: **In this cross-sectional study, 88 children diagnosed with cirrhosis were included, and the values of hemoglobin, serum iron levels and relationship between serum iron (SI), total iron-binding capacity (TIBC), prothrombine time (PT), international normalization ratio (INR), total and direct bilirubin and hepatic enzymes were estimated using paired *t* test, Mann-Whitney, Chi-square and Kruskal-Wallis tests.

**Results: **Forty-six (52.3%) of 88 children were girls and 42 (47.7%) were boys. Forty-eight (54.5%) patients had anemia and 8 (9%) had iron deficiency anemia (5 boys, 5.6%, and 3 girls, 3.4%). No relationships were observed between iron deficiency anemia and the patient’s age or gender, whereas there was a relationship between iron deficiency and severity and duration of the disease, although the correlation was not statistically significant.

**Conclusion:** The high frequency of iron deficiency anemia in children with cirrhosis (9%) suggests that timely screening should be used for early diagnosis and treatment.

## Introduction

 Anemia of chronic disease is the most common form of anemia in outpatients. This type of anemia is similar in appearance to iron deficiency anemia (IDA) but is mainly caused by the entrapment of iron in mononuclear phagocytic cells of the reticuloendothelial system due to inflammation.^1^ Serum iron is usually low, and with normochromic-normocytic erythrocytes or in similar cases of IDA, microcytosis and hypochromia is evident. High bone marrow iron storage, normal or elevated ferritin and decreased TIBC are features that distinguish it from IDA.^2^^-^^4^ In anemia of chronic disease, hepcidin concentration in response to the inflammatory cytokine interleukin 6 is increased.^5^ Hepcidine inhibits ferroportin by binding and internalizing it within the cell. In turn, ferroportin inhibition stops the transfer of iron from mononuclear phagocytes to erythroid precursors. As a result, iron is retained within cells and plasma iron levels are reduced.^6^

In chronic inflammation, the compensatory increase in erythropoietin is not commensurate with the severity of anemia. Other reasonable explanations of iron trapping in chronic inflammatory disorders remain unconfirmed. Iron trapping may inhibit the iron-dependent growth of microorganisms or enhance some aspects of host immunity. Although iron and erythropoietin prescription can transiently correct anemia, treatment of the underlying disease will improve anemia.^2^^-^^4^ Decreased SI and TIBC, along with normal or elevated ferritin levels, are helpful in differentiating anemia of chronic disease from IDA. Transferrin receptor level is the most useful factor in distinguishing between the two types of anemia, because it is not associated with inflammation. Serum transferrin receptor concentration is increased in IDA, but is normal in anemia of chronic disease. Transferrin saturation level is below normal in IDA.^7^^, ^^8^

 In patients with cirrhosis, anemia can develop from several factors. Destruction of red blood cells in the spleen is considerable, and blood loss is also frequently caused by damage to the stomach, duodenum or hemorrhoids and tearing of esophageal varices. Red blood cell production is reduced because of excessive blood loss and secondary body iron depletion. Decreased erythropoietin levels also curtail red blood cell production by the bone marrow.^9^^,^^10^ Patients with cirrhosis are at risk of developing both IDA and anemia of chronic disease concomitantly, and the adverse effects on health-related quality of life in these patients are evident.^10^ Because it is easier to correct IDA than anemia of chronic disease, the diagnosis and treatment of IDA can reduce the need for other diagnostic tests and improve the quality of life in patients with cirrhosis. The aim of this study was to evaluate prevalence of iron deficiency anemia in children with cirrhosis. 

## SUBJECTS AND METHODS

 In this cross-sectional study, 88 patients less than 18 years old with clinically, radiologically and histologically-confirmed liver cirrhosis were enrolled. All patients with acute liver diseases were excluded. Written informed consent was obtained from the patients or their parents. The Shiraz University of Medical Sciences Ethics Committee approved this study. 

μμPT (manual method), INR, total and direct bilirubin (mg/dL), aspartate aminotransferase (AST) and alanine aminotransferase (ALT) (U/L) (Prestige 24i automated analyzer, Casmos Biomedical Ltd, UK). These results were used to calculate transferrin saturation (TIBC/SI).

Iron deficiency anemia was defined as a hemoglobin level less than 2 standard deviations for age and sex and transferrin saturation less than 20%. The clinical status of the children was assessed with the Pediatric End stage Liver Disease (PELD) score and Child-Pugh score. The PELD score is calculated based on age, growth status, INR, total bilirubin and serum albumin. The parameters used in the Child-Pugh score are serum albumin, total bilirubin, PT, INR, ascites and hepatic encephalopathy. We also investigated the relationship between IDA and sex, age, liver disease stage, disease duration, total bilirubin, albumin and PT/INR separately. 

Statistical analyses were done with SPSS V 15 software (Chicago, IL, USA) with the paired *t* test, Mann-Whitney, Chi-square and Kruskal-Wallis tests. A p-value of <0.05 was considered statistically significant. 

## Results

 The most common causes of cirrhosis in our patients were biliary atresia (21.5%), progressive familial intrahepatic cholestasis (17%), cryptogenic hepatitis (11.5%), autoimmune hepatitis (11.5%) and idiopathic neonatal hepatitis (5.5%). Other causes of cirrhosis were cystic fibrosis, Budd Chiari syndrome and thyrosinemia. Mean hemoglobin level (± standard deviation) was 11.45 ± 3.07 g/dL (range 4.5 - 19.0 g/dL) and mean corpuscular volume was 83 ± 5.5 fL (range 57.4-114.0 fL). Mean SI was and 55 ± 25.4 (range 8-171) and mean TIBC were 365 ± 60 μg/dL (range 238-594 μg/dL) (Table1). 

A total of 48 (54.54%) patients were diagnosed as having anemia. Eight (9%) of these patients (5 males, 5.6% and 3 females, 3.4%) had IDA. The difference between the sexes in the prevalence of cirrhosis was not statistically significant (p=0.57). Likewise, the difference between the sexes was not statistically significant in patients with cirrhosis who also had IDA. (p=0.3). 

Although patients with cirrhosis who had IDA were younger than patients who did not have this type of anemia (4.9 years vs 6.9 years), there was no relationship between age and IDA in these patients (p=0.23). Among all 88 patients with cirrhosis, 13 (14.8%) had low transferrin saturation, and 8 patients (9.1%) had IDA. 

In the present study, there was no significant relationship between IDA and disease duration, although mean duration of the disease in patients with IDA was shorter than in patients without this type of anemia (1.5 ± 1.67 vs. 2.0 ± 2.78 years, p=0.34). 

The most common causes of cirrhosis in patients with IDA were biliary atresia, neonatal hepatitis and hepatitis B. There was no significant relationship between the cause of cirrhosis and IDA (p=0.48). Although the mean levels of liver enzymes, albumin, bilirubin, PT, INR and platelets in patients with IDA were lower than in patients without IDA, the differences were not statistically significant (p=0.68 ). 

Because INR was normal in patients with IDA, it was not possible to compare the relationships of INR level with SI or TIBC. There were no significant relationships between SI, TIBC and total bilirubin and albumin levels. The PELD score was not significantly higher in children who had IDA, compared to those who did not have this type of anemia (p=0.29). Child-Pugh score did not differ significantly between groups. However, the PELD score increased with decreasing SI (p=0.007). Mean serum albumin concentration was 3.8 ± 0.71 g/dL in patients with anemia and 4.06 ± 0.8 g/dL in patients who did not have anemia (p=0.31).

## Discussion

 Cirrhosis has a high rate of mortality and is associated with various complications and complex, long-term treatment. Although IDA is a distinguishing feature in chronic liver disease, it has received relatively little attention. This type of anemia can lead to hemodynamic abnormalities such as increased cardiac output and mean arterial pressure, and decreased systemic vascular resistance, especially in cirrhosis.^10^ We found IDA in 9% of the children with cirrhosis in the present study which was higher than all the other studies; this relatively high figure was probably be due to complications of cirrhosis such as esophageal varices, coagulation abnormalities and impaired intestinal iron absorption. In a study by Haghbin et al. of 260 school-aged children in Iran, 4.5% were identified as having IDA based on a hemoglobin concentration less than 12 g/dL and transferrin saturation less than 15%.^11^ Mean Hb level was lower in our study than in the study by Haghbin et al. but mean SI and TIBC were within similar ranges.^11^ Another study in Iran by Fesharakinia et al. showed that the prevalence of IDA in school-aged children was 1.8%, and IDA was significantly correlated with female sex, menstruation and age. ^13^ In another study in Fars Province, Iran by Ramzi et al on 363 adolescent school girls, the prevalence of anemia and IDA were 5.8% and1.7%, respectively ^14^ which is lower than the frequency of IDA in our patients. 

In end stage liver disease, the risk of IDA increases with increasing severity of the disease (PELD score) and its complications, especially coagulopathy. In the present study; however, this relationship was not statistically significant with increasing PELD score, although SI decreased, and this relationship was statistically significant. 

Our study showed, although the mean Child score was lower in iron deficient children than non-iron deficient patients, but this relationship was not statistically significant. A study by Cirera and colleagues reported a significant relationship between severity of disease (Child score) and frequency of anemia.^9^

Our finding implies that children with a high Child score may be more likely to develop early IDA. Similar results were seen in a study by Maheshwari et al. in transplant patients.^12^ In their study, the frequency of IDA decreased with increasing post-transplant time; this finding may be due to improvements in the complications of cirrhosis. Their study also reported that patients with IDA were, on average, younger than patients without IDA;^11^ because the risk of IDA in healthy children is higher at younger ages. 

In the present study, mean serum albumin concentration did not differ significantly between patients with and without anemia.

**Table1:** Variables characteristics of patients with cirrhosis with and without anemia

	**Cirrhosis with anemia** **(Mean ** **±** ** SD)**	**Cirrhosis without ** **anemia(Mean** ** ±** ** SD)**	**P value**
**Age (year)**	4.9 ± 5.19	6.9 ± 4.5	
**Duration (year)**	1.5 ± 1.67	2 ± 2.78	0.34
**Total protein (gm/dL)**	7.42 ± 0.69	8.08 ± 1.08	0.4
**Globulin (gm/dL)**	3.61 ± 0.72	3.9 ± 1.09	0.56
**Albumin (gm/dL)**	3.87 ± 0.71	4.06 ± 0.8	0.31
**AST (IU/L)**	206.25 ± 149.66	211.59 ± 256	0.59
**ALT (IU/L)**	107.12 ± 80.74	141.92 ± 210.86	0.43
**Total bilirubin (mg/dL)**	7.83 ± 9.52	10.01 ± 11.56	0.88
**Direct bilirubin (mg/dL)**	4.4 ± 5.65	3.56 ± 4.22	0.07
**PT (second)**	14.87 ± 5.4	15.23 ± 4.22	0.83
**INR**	1.14 ± 0.2	1.64 ± 0.92	0.08
**Platelet (/mcL)** **Serum iron (mcg/dL)** **TIBC (mcg/dL)** **Child Score** **PELD Score**	201657 ± 6866 61.8 ± 47.8 357 ± 102.7 7.63 ± 2.38 17.87 ± 12.58	210163 ± 4593 74.5 ± 43.9 359.3 ± 80.2 7.71 ± 2 11.4 ± 9.5	0.86 0.205 0.994 0.45 0.29

**SD: **standard deviation, **AST: **Aspartate Aminotransferase, **ALT: **Alanine Transaminase, **PT: **Prothrombin Time, **INR:** International Normalized Ratio, **TIBC:** Total iron binding capacity, **PELD score:** Pediatric end-stage liver disease score

Cirera et al. reported a significant relationship between severity of disease and frequency of anemia.^9^

Our finding implies that children with a high Child score may be more likely to develop early IDA. Similar results were seen in a study by Maheshwari et al. in transplant patients.^12^ In their study, the frequency of IDA decreased with increasing post-transplant time; this finding may be due to improvements in the complications of cirrhosis. Their study also reported that patients with IDA were, on average, younger than patients without IDA;^11^ because the risk of IDA in healthy children is higher at younger ages. 

In the present study, mean serum albumin concentration did not differ significantly between patients with and without anemia. Cirera et al. however, found a significant relationship between anemia and serum albumin. In both studies, mean serum albumin in patients with anemia was lower than in patients without anemia. The different results obtained in these two studies may be due to the small sample size we used. Further studies with larger patient samples are recommended to shed light on the possible relationship between these two factors. 

The current study, like the study conducted by Cirera et al. showed that mean liver enzymes and PT were lower in patients with anemia than patients without anemia. Unlike the results of the study conducted by Cirera et al. no statistically significant difference was observed between the two study goups.^9^

Nacoulma et al. found hypochromic microcytic anemia in 20% of patients with liver cirrhosis; these authors concluded that anemia was not exclusively linked to blood loss or iron deficiency. They also emphasized the importance of accurately interpreting the hemogram to avoid routine iron supplement prescription.^15^ As patients do not always present with classical laboratory findings and may have more than one cause of anemia, a detailed and cautious approach to diagnosis should be considered in patients with hepatic disorders who also have anemia.^16^

## CONCLUSION

 In this study, the frequency of low hemoglobin level and IDA in patients with cirrhosis was 54.5% and 9%, respectively. As patients with severe liver disease will more likely to develop IDA at a younger age, we recommend appropriate early screening for patients with cirrhosis to detect anemia. Iron supplementation should be prescribed for patients with cirrhosis who are at risk of developing IDA, and for patients who already have IDA, especially those with more severe disease.
